# Supplementary material for: Identification and characterization of a novel enhancer in the HTLV-1 proviral genome
Source: Nat Commun. 2022 May 3;13:2405. doi: 10.1038/s41467-022-30029-9 (PMC9065021; doi:10.1038/s41467-022-30029-9)
Supplement: Supplementary file 1 — Supplementary Information [file 41467_2022_30029_MOESM1_ESM.pdf]

Supplementary Information File

Identification and characterization of a novel enhancer in the HTLV-1 proviral genome

Misaki Matsuo[1,2], Takaharu Ueno[3#], Kazuaki Monde[4#], Kenji Sugata[1#], Benjy Jek Yang Tan[1,2], Akhinur Rahman[1], Paola Miyazato[1,2], Kyosuke Uchiyama[1,2], Saiful Islam[1,2], Hiroo Katsuya[1,5], Shinsuke Nakajima[3], Masahito Tokunaga[6], Kisato Nosaka[7,8], Hiroyuki Hata[9], Atae Utsunomiya[6,10], Jun-ichi Fujisawa[3], Yorifumi Satou[1,2]*

[1] Division of Genomics and Transcriptomics, Joint Research Center for Human Retrovirus Infection, Kumamoto University, Kumamoto, 860-8556, Japan.
[2] International Research Center for Medical Sciences (IRCMS), Kumamoto University, Kumamoto, 860-0811Japan.
[3] Department of Microbiology, Kansai Medical University, Osaka, 573-1010, Japan.
[4] Department of Microbiology, Faculty of Life Sciences, Kumamoto University, Kumamoto, 860-8556, Japan.
[5] Division of Hematology, Respiratory Medicine and Oncology, Saga University, Saga, 849-8501, Japan.
[6] Department of Hematology, Imamura General Hospital, Kagoshima, 890-0064, Japan.
[7] Department of Hematology, Rheumatology and Infectious Disease, Kumamoto University Hospital, Kumamoto, 860-8556, Japan.
[8] Cancer Center, Kumamoto University Hospital, Kumamoto, 860-8556, Japan.
[9] Division of Informative Clinical Sciences, Faculty of Life Sciences, Kumamoto University, Kumamoto, 862-0972, Japan.
[10] Graduate School of Medical and Dental Sciences, Kagoshima University, Kagoshima, 890-8544, Japan.

[#]These authors contributed equally: Takaharu Ueno, Kazuaki Monde, Kenji Sugata.

*Correspondence and requests for materials should be addressed to Y.S.
1-1-1 Honjo, Chuo-ku, Kumamoto
860-8556 Japan
Tel: +81-96-373-6830
E-mail: y-satou@kumamoto-u.ac.jp

Supplementary Fig. 1

a

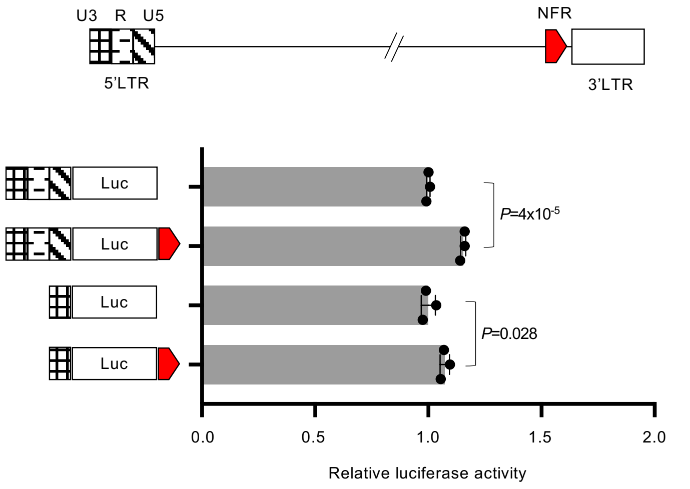

b

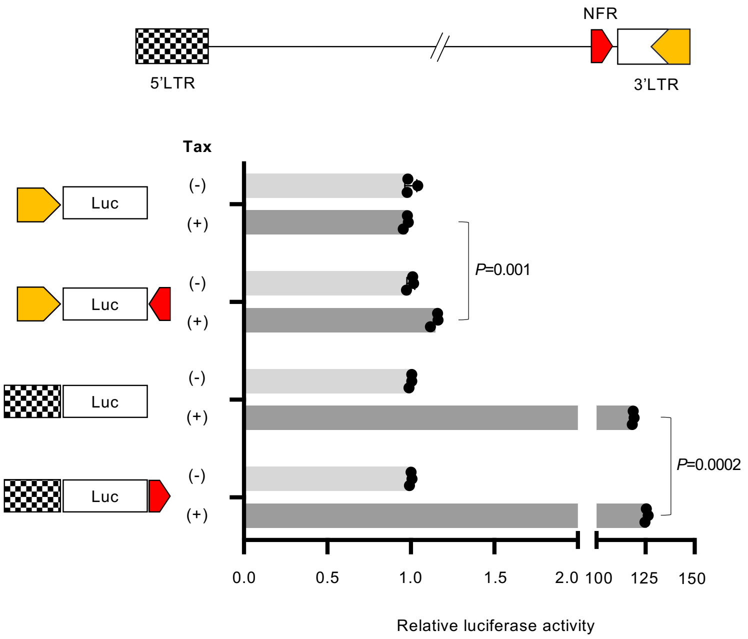

Supplementary Figure 1. Transcriptional regulatory function of the NFR for the 5'LTR under the Tax expression. a Top panel indicates schematic figure of the HTLV-1 provirus structure. NFR and parts of 5'LTR divided into U3, R, U5 are shown as red, lattice pattern, dotted-line pattern, diagonal stripe pattern, respectively. Transcriptional regulatory function of the NFR was analyzed by luciferase reporter assays in Jurkat cells. Whole 5'LTR (upper) or U3 region (lower) were used as a promoter. Luciferase activity was normalized to Renilla activity. Relative luciferase activity is defined as the fold change to pGL4-basic-whole 5'LTR (upper) or U3 region (lower). *n* = 3 biologically independent samples, mean ± SD. *P* values are calculated by two-sided Student's *t*-test. **b** Top panel indicates schematic figure of the HTLV-1 provirus structure. 5'LTR, HBZ promoter and NFR are shown as black plaid, yellow and red, respectively. Transcriptional regulatory function of the NFR was analyzed by luciferase reporter assays with or without Tax expression in Jurkat cells. The HBZ promoter (upper) or 5'LTR (lower) were used as a promoter. Luciferase activity was normalized to Renilla activity. Relative luciferase activity is defined as the fold change to without Tax expression for each reporter vector. *n* = 3 biologically independent samples, mean ± SD. *P* values are calculated by two-sided Student's *t*-test.

Supplementary Fig. 2

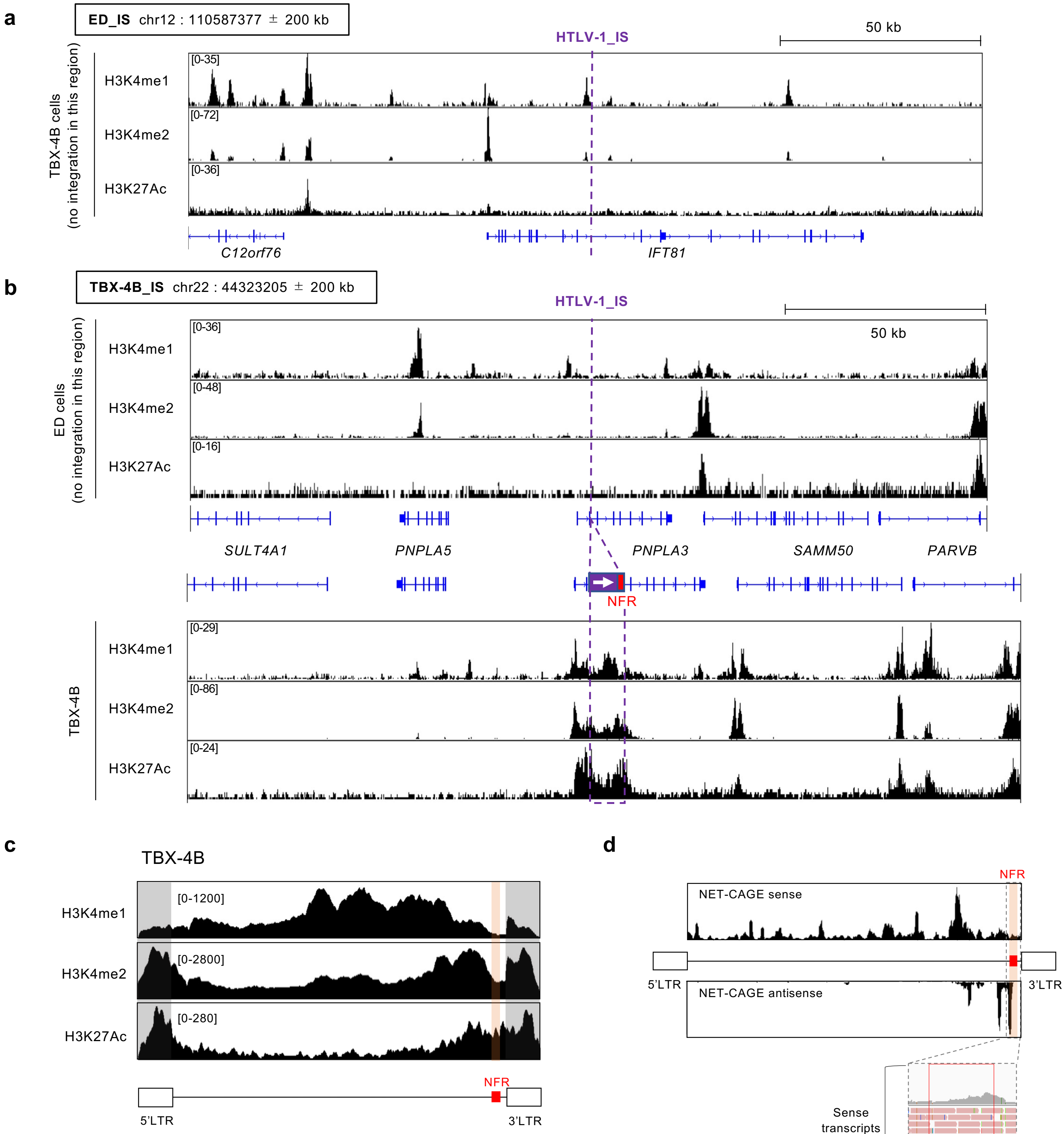

Supplementary Figure 2. The nucleosome-free region harbors enhancer-related histone modifications and produces enhancer RNAs in TBX-4B cells. a H3K4me1 (top row), H3K4me2 (middle row), and H3K27Ac (bottom row) occupancy near the viral integration site of ED cells. ChIP-seq signals of TBX-4B cells, which do not have viral integration in this region, were visualized by IGV. **b** H3K4me1 (top row), H3K4me2 (middle row), and H3K27Ac (bottom row) occupancy near the viral integration site of TBX-4B cells. ChIP-seq signals of ED cells (upper panel), which do not have viral integration in this region, and TBX-4B cells (lower panel) were visualized by IGV. The location (purple square) of provirus and NFR (red square) in TBX-4B cells are shown. The white arrow represents the direction of integrated provirus. The range in square brackets indicates the range of read counts. **c** H3K4me1 (top row), H3K4me2 (middle row), and H3K27Ac (bottom row) occupancy within the provirus in TBX-4B cells. ChIP-seq signals were visualized by IGV. Gray-shaded areas and orange-shaded area indicate the ChIP-seq signals mapped to LTRs and NFR, respectively. The range in square brackets indicates the range of read counts. **d** NET-CAGE results of TBX-4B cells in the sense (top row) and antisense (bottom row) orientations. The bottom panel is an enlarged image of the signals around the NFR. NET-CAGE signals were visualized by IGV.

Supplementary Fig. 3

a

NFR sequences	SRF mut sequence		ELK-1 mut sequence		SRF mut sequence	SRF mut sequence
wt	CCTTATTTGGACA	TTTACCGATGGCACGCCTATG	ATTTCCGGGCCC	TG	CCCTAAAGATGGCC	AGCCATCTTTAGTA
mut_1	ACTCATCTGGACG	TTTACCGATGGCACGCCTATG	ATTTCCGGGCCC	TG	TCCAAAGGACGGTC	AACCCTCGCTCGTG
mut_2	CCTTATTTGGACA	TTTACCGATGGCACGCCTATG	ATCAGTGGCCCA	TG	CCCTAAAGATGGCC	AGCCATCTTTAGTA
mut_3	ACTCATCTGGACG	TTTACCGATGGCACGCCTATG	ATCAGTGGCCCA	TG	TCCAAAGGACGGTC	AACCCTCGCTCGTG

b

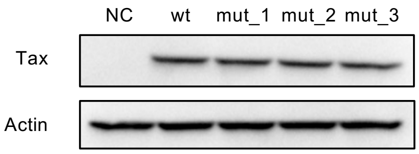

Supplementary Figure 3. HTLV-1 mutants lacking SRF/ELK-1 binding sites show comparable expression of Tax. a Nucleotide sequences shows that the mutations introduced to generate 3 different NFR mutants shown in Fig. 3d. The mutated nucleotides are shown in color as red. **b** Tax protein levels in nuclear lysates of 293T cells transfected with wt or enhancer-region-mutated Tax-expression vectors. Data shown are representative of two independent experiments.

Supplementary Fig. 4

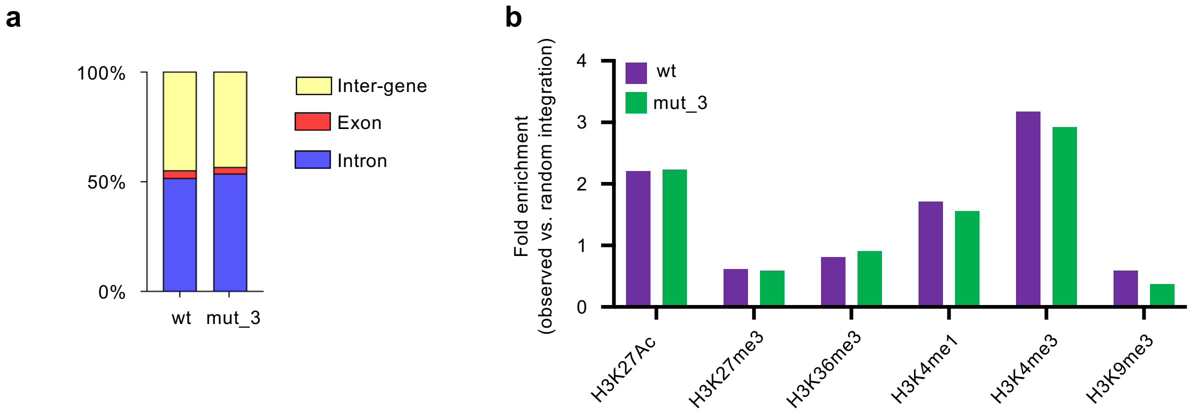

Supplementary Figure 4. Relationship between HTLV-1 ISs and genetic/epigenetic environment in JET cells infected with HTLV-1-wt or mut_3. a The frequency of ISs within genes or inter-genes in JET cells infected with HTLV-1-wt or mut_3. **b** Fold enrichment of IS distribution in each histone modification compared to random distributions in cells infected with each molecular clone.

Supplementary Fig. 5

a

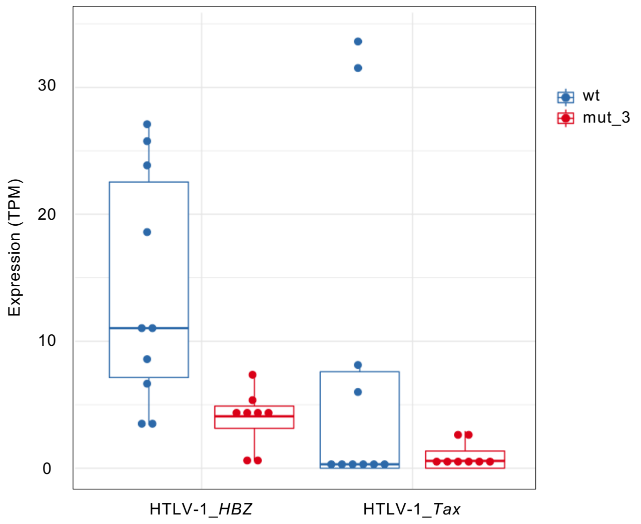

b

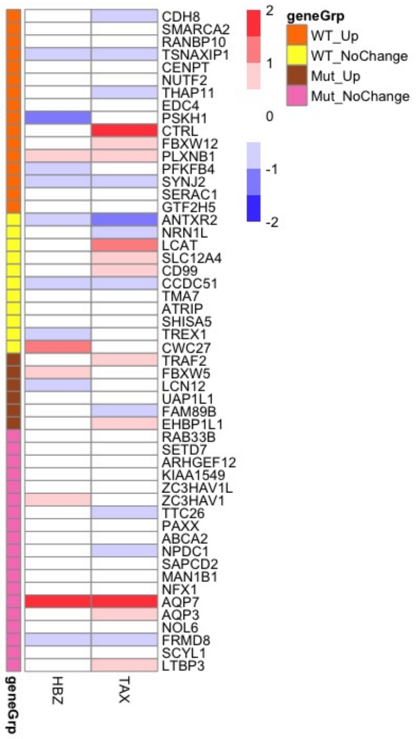

Supplementary Figure 5. Characterization of viral and host gene expression in JET cells infected with HTLV-1-wt or mut_3. a Expression level of *HBZ* or *Tax* transcripts in JET cells infected with HTLV-1-wt or mut_3 (See Supplementary Table 2). Expression data is shown as transcripts per million (TPM) obtained from bulk mRNA-seq for each clone in duplicates. n = 8-10 biologically independent samples. Box plots represent the median and interquartile range (IQR) while the whiskers extend up to the minimum and maximum values. **b** We analyzed publicly available RNA-seq data regarding Jurkat cell with inducible *Tax* or *HBZ* gene and show fold changes induced by *HBZ* or *Tax* expression as Heatmap graph. The genes listed are host genes found near the ISs in JET cells infected with HTLV-1-wt or mut_3 in Fig. 5e (Also see Supplementary Table 2).

Supplementary Fig. 6

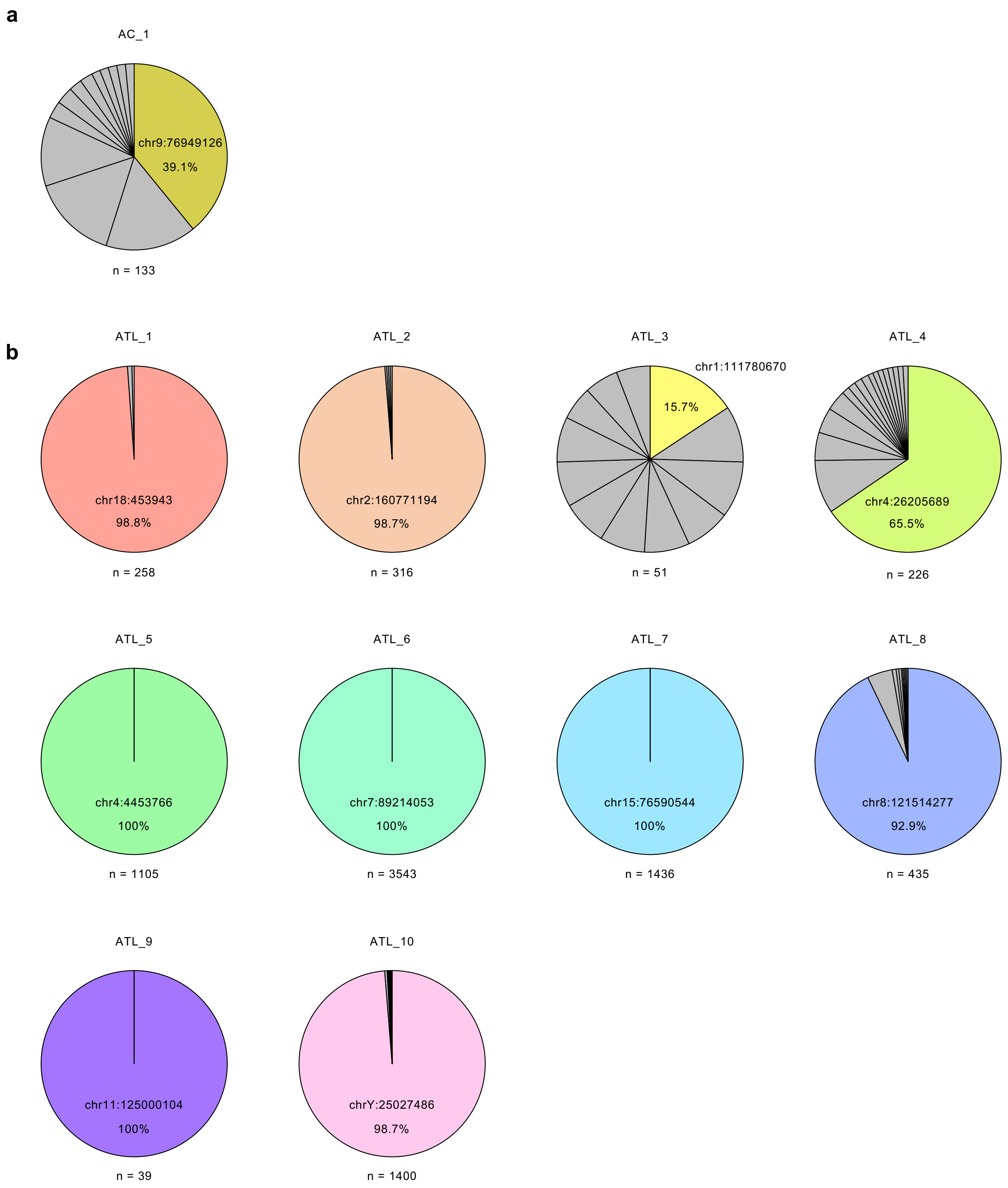

Supplementary Figure 6. Clonality of HTLV-1-infected clones in each individual. Pie charts show clonal abundance of each clone in each individual, (**a**) one AC and (**b**) ten ATL cases in this study. Clonality was evaluated by DNA-capture-seq or Ligation-mediated-PCR as described previously[15]. The numbers of total cells investigated are shown below each pie chart.

Supplementary Fig. 7

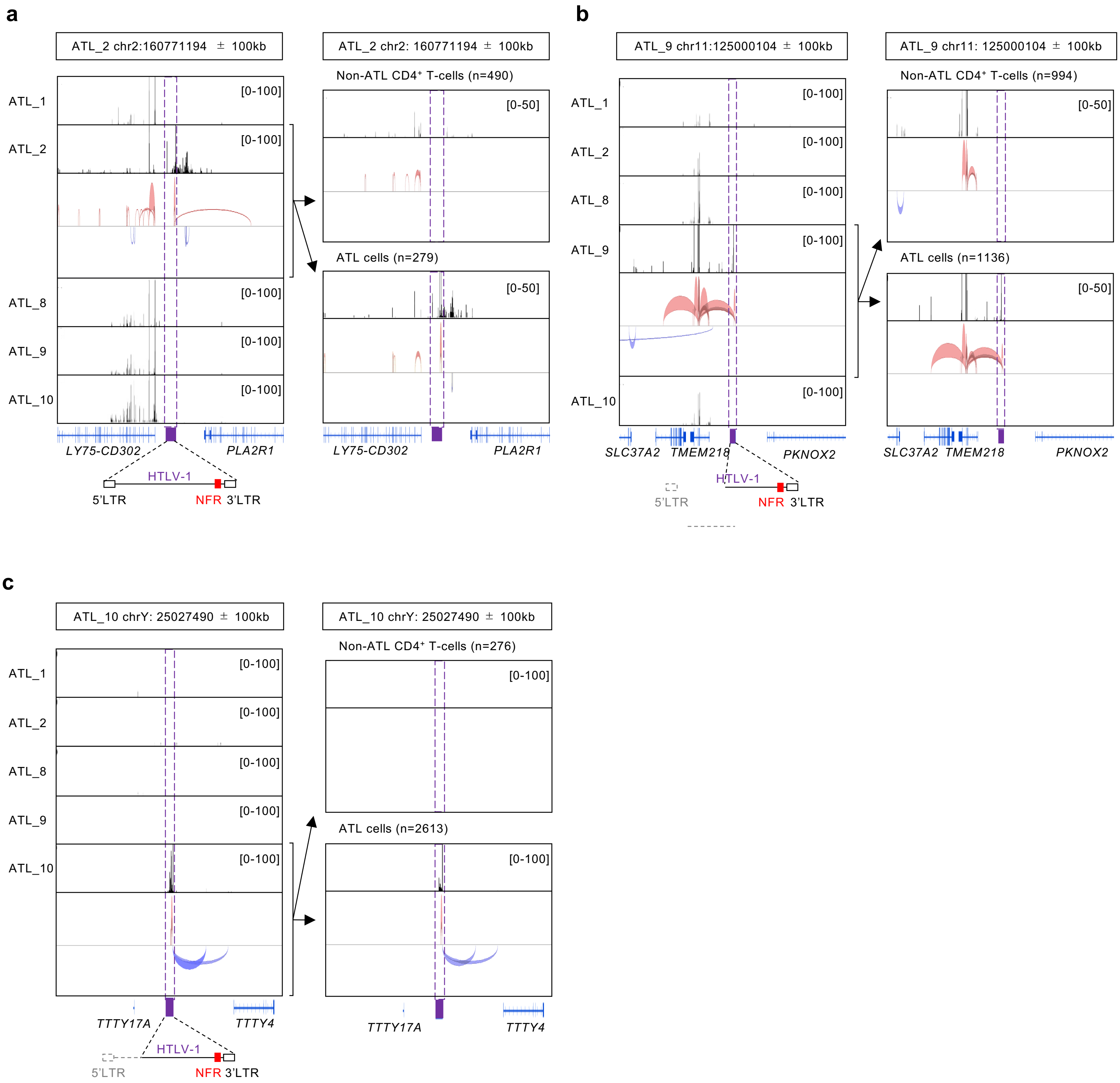

Supplementary Figure 7. Transcriptional characterization of the provirus and the flanking host genomes in freshly isolated PBMCs from infected individuals. a-c Local transcriptome including viral integration site are visualized by IGV. We obtained scRNA-seq data from five ATL cases. The data shown were region with viral IS of (**a**) ATL_2, (**b**) ATL_9 and (**c**) ATL_10, respectively. Data from CD4⁺ T-cells are shown in the left panel. CD4⁺ T-cells are further divided into non-ATL CD4⁺ T-cells (right, upper panel) and ATL cells (right, lower panel).

Supplementary Fig. 8

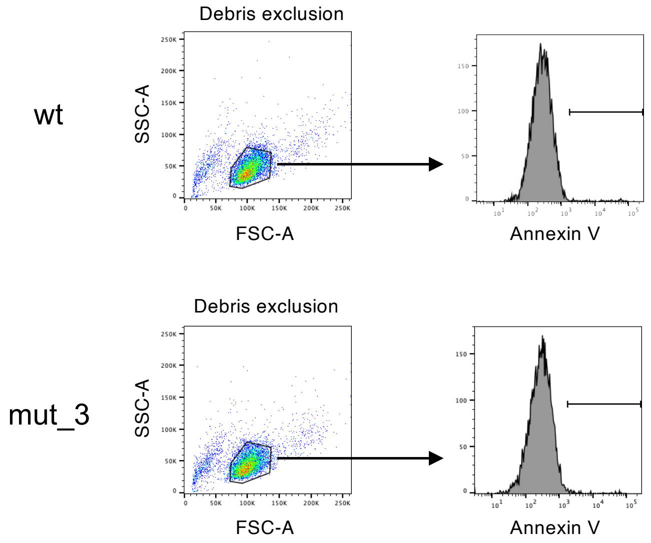

Supplementary Figure 8. Gating strategy for Fig.4j. We gated alive cell population to exclude debris at FSC/SCC dot plots and measured Annexin V positive cells as apoptotic cells. The percentages of apoptotic cells in the alive cell population of JET cells infected with HTLV-1-wt or mut_3 were shown as bar graphs in Fig. 4j. Each dot represents a cell and the color indicates cell poopulation density from least dense (blue) to most dense (red).

Supplementary Table 1. The profile of HTLV-1-infected patients' samples.

Patient ID	Disease type	PVL (%)	IS		Strand	Provirus type	Clonal abundance (%)
AC_1	Asymptomatic carrier	15.04	chr9:	76949126	+	5'defective	39.1
			chr10:	42376989	-	full	15.8
			chr8:	133538142	+	full	15
			chr13:	76575759	+	full	12
			chr9:	20647317	+	full	3
ATL_1	Chronic ATL	43.64	chr18:	453943	-	full	98.8
ATL_2	Smoldering ATL	58.75	chr2:	160771194	+	full	98.7
ATL_3	Smoldering ATL	8.9	chr1:	111780670	+	5'defective	15.7
			chr3:	142210501	+	full	9.8
			chr10:	90920211	+	5'defective	9.8
			chr2:	116659472	+	full	7.8
			chr4:	28737000	-	full	7.8
ATL_4	Acute ATL	25.4	chr4:	26205689	-	full	65.5
			chr1:	172317745	-	full	9.3
			chrY:	8327809	-	full	4.9
			chr19:	21651030	+	full	4.4
			chr1:	28286950	-	full	3.5
ATL_5	Acute ATL	102	chr4:	4453766	-	full	100
ATL_6	Smoldering ATL	121.6	chr7:	89214053	+	5'defective	100
ATL_7	Chronic ATL	62.7	chr15:	76590544	-	full	100
ATL_8	Chronic ATL	23.8	chr8:	121514277	-	5'defective	92.9
ATL_9	Chronic ATL	33.4	chr11:	125000104	+	5'defective	100
ATL_10	Chronic ATL	66.56	chrY:	25027486	+	5'defective	98.7

Supplementary Table 1. The profile of HTLV-1-infected patient samples.
The disease type, proviral load, integration site, the strand direction and provirus type are shown.

Supplementary Table 2. The profile of HTLV-1-wt- and -mut_3-infected clones.

Clone name	Integration site		Proviral direction in the host genome
wt_#1	chr11:	92526218	+
	chrX:	2718939	−
wt_#2	chr2:	36362768	−
	chr3:	48487187	+
	chr6:	158528725	−
	chr8:	21638241	−
wt_#3	chr4:	80736011	−
	chr16:	61845376	+
wt_#4	chr9:	2233717	−
	chr16:	60365280	+
	chr16:	67905552	+
wt_#5	chr5:	64256335	+
mut_3_#1	chr4:	140454610	−
	chr11:	120298441	−
mut_3_#2	chr2:	60025417	+
mut_3_#3	chr7:	138733748	+
	chr9:	139894554	+
mut_3_#4	chr9:	33403846	+
	chr11:	65253493	−

Supplementary Table 2. The profile of HTLV-1-wt and -mut_3-infected clones.
The integration site and the strand direction of HTLV-1-wt-infected clones and mut_3-infected clones are shown.

Supplementary Table 3. Primers and construct sequence for luciferase reporter assay.

Primers	Sequence
XhoI-3'LTR300-F	5’-ctcgagTGTGTACTAAATTTCTCTCCTGGA-3’
HindIII-3'LTR300-R	5’-aagcttGCGTCCGCCGTCTAGGTAAGTT-3’
XhoI-5'LTR-F	5’-ctcgagTGACAATGACCATGAGCCCCAA-3’
HindIII-5'LTR-R	5’-aagcttTGTGTACTAAATTTCTCTCCTGGA-3’
BamHI-NFR-F	5’-ggatccTCCTTCCGTTCCACTCAAC-3’
BamHI-NFR-R	5’-ggatccTGGTAGGCCTTGGTTTGA-3’
NFR_mutant_construct	5’-<br>TCCTTCCGTTCCACTCAACCCTCACCACTCCAGGACTCATCTG<br>GACGTTTACCGATGGCACGCCTATGATCAGTGGCCCATGTCC<br>AAAGGACGGTCAACCCTCGCTCGTGCTACAGTCCTCCTCCTT<br>TATATTTCACAAATTTCAAACCAAGGCCTACCA<br>-3’
NFR-CTCF_Gibson-Assembly-F	5’-<br>GAGTGGTGAGGGTTGAGTGGAACGGAAGGAACAAGGAGGA<br>GGAGGAAGCTGTGCTTGAC<br>-3’
NFR-CTCF_Gibson-Assembly-R	5’-<br>GTCAAGCACAGCTTCCTCCTCCTCCTTGTTCCTTCCGTTCCAC<br>TCAACCCTCACCACTC<br>-3’
NFR_Gibson-Assembly-F	5’-<br>AAATGTGGTAAAATCGATAAGGATCCTGGTAGGCCTTGGTTT<br>GAAATTTGT<br>-3’
NFR_Gibson-Assembly-R	5’-<br>GAAGGCTCTCAAGGGCATCGGTCGACTGGTAGGCCTTGGTTT<br>GAAATTTGT<br>-3’
CTCF_Gibson-Assembly-F	5’-<br>AATGTGGTAAAATCGATAAGGATCCCTGCTCTTCCTGCTTCC<br>TCCTGGCGA<br>-3’
CTCF_Gibson-Assembly-R	5’-<br>AAGGCTCTCAAGGGCATCGGTCGACCTGCTCTTCCTGCTTCC<br>TCCTGGCGA<br>-3’

Supplementary Table 3. Primers and construct sequence for luciferase reporter assay.
The sequences of primers used for each reporter construct and the NFR mutant construct.

Supplementary Table 4. Oligonucleotides for qRT-PCR and PVL measurement.

Primers and probes	Sequence
qRT-PCR_HBZ-F	5’-GGACGCAGTTCAGGAGGCAC-3’
qRT-PCR_HBZ-R	5’-CCTCCAAGGATAATAGCCCG-3’
qRT-PCR_tax-F	5’-CCGGCGCTGCTCTCATCCCGGT-3’
qRT-PCR_tax-R	5’-GGCCGAACATAGTCCCCCAGAG-3’
ddPCR_tax-F	5’-CGGATACCCAGTCTACGTGTT-3’
ddPCR_tax-R	5’-CAGTAGGGCGTGACGATGTA-3’
ddPCR_alb-F	5’-TGCATGAGAAAACGCCAGTAA-3’
ddPCR_alb-R	5’-ATGGTCGCCTGTTCACCAA-3’
ddPCR_tax-probe	5’-/ 56-FAM/CTGTGTACA/ZEN/AGGCGACTGCC/3IABkFQ/ -3’
ddPCR_alb-probe	5’-/5HEX/TGACAGAGT/ZEN/CACCAAATGCTGCACAGAA/3lABkFQ/ -3’

Supplementary Table 4. Oligonucleotides for qRT-PCR and PVL measurement.
The sequences of primers and probes for qRT-PCR and PVL measurement.

Supplementary Table 5. Labeled and non-labeled probe sequences for EMSA

Probes	Sequence
Biotin_wt-probe	5'-<br>bio_ACTCAACCCTCACCACTCCAGGCCTTATTTGGACATTTACC<br>GATGGCACGCCTATGATTTCCGGGCCCTGCCCTAAAGATGGCC<br>AGCCATCTTTAGTACTACAGTCCTCCTCCTTTATATT<br>-3'
Biotin_NC3100-probe	5'-<br>bio_CTACTACTACTCTCAGAGGCCACAATGGCTTCCCTAATCTC<br>CCATGGGTTGCCTGTGTCCGAAAACAAAACCCAGCAAACCCCT<br>GGAACAATTAAGTTCCTAGGGCAGATAATTTCACCCA<br>-3'
Biotin_NC5000-probe	5'-<br>bio_CTGGTCTTAATAGCCGCCAGTGGAAAGGACCACAGGAGG<br>CTCTCCAAGAAGCTGCCGGCGCTGCTCTCATCCCGGTAAGCGC<br>TAGTTCTGCCCAGTGGATCCCGTGGAGACTCCTCAAGCG<br>-3'
wt_unlabeled competitor-probe	5'-<br>ACTCAACCCTCACCACTCCAGGCCTTATTTGGACATTTACCGA<br>TGGCACGCCTATGATTTCCGGGCCCTGCCCTAAAGATGGCCAG<br>CCATCTTTAGTACTACAGTCCTCCTCCTTTATATT<br>-3'
mut1_ unlabeled competitor-probe	5'-<br>ACTCAACCCTCACCACTCCAGGACTCATCTGGACGTTTACCGA<br>TGGCACGCCTATGATTTCCGGGCCCTGTCCAAAGGACGGTCAA<br>CCCTCGCTCGTGCTACAGTCCTCCTCCTTTATATT<br>-3'
mut2_ unlabeled competitor-probe	5'-<br>ACTCAACCCTCACCACTCCAGGCCTTATTTGGACATTTACCGA<br>TGGCACGCCTATGATCAGTGGCCCATGCCCTAAAGATGGCCAG<br>CCATCTTTAGTACTACAGTCCTCCTCCTTTATATT<br>-3'
mut3_ unlabeled competitor-probe	5'-<br>ACTCAACCCTCACCACTCCAGGACTCATCTGGACGTTTACCGA<br>TGGCACGCCTATGATCAGTGGCCCATGTCCAAAGGACGGTCA<br>ACCCTCGCTCGTGCTACAGTCCTCCTCCTTTATATT<br>-3'

Supplementary Table 5. Labeled and non-labeled probe sequences for EMSA.
The sequences of labeled and non-labeled probes for EMSA are described.

Supplementary Table 6. Primers for MNase assay

Primers	Sequence
MNase-5’LTR_j-F	5’-GACAGCCCATCCTATAGCACTC-3’
MNase-5’LTR_j-R	5’-CTAGCGCTACGGGAAAAGATT-3’
MNase-gag-F	5’-CAGAGGAAGATGCCCTCCTATT-3’
MNase-gag-R	5’-GTCAACCTGGGCTTTAATTACG-3’
MNase-pol-F	5’-TTCCGCCACCGCACAAGTCG-3’
MNase-pol-R	5’-TGCCTTGGAAGGTGCCCAGG-3’
MNase-env-F	5’-CTGTTCCCACCCTAGGATCCCG-3’
MNase-env-R	5’-GAGGCTCTTTCCTGAGGCGAGG-3’
MNase-NFR-F	5’-CTCCTTCCGTTCCACTCAAC-3’
MNase-NFR-R	5’-GTGGTAGGCCTTGGTTTGAA-3’
MNase-3’LTR_j-F	5’-AATACACCAACATCCCCATTTC-3’
MNase-3’LTR_j-R	5’-GTTTTTCACTGGGAGGCTCTAA-3’

Supplementary Table 6. Primers for MNase assay.
The sequences of primers for MNase assay are described.

Supplementary Table 7. Oligonucleotides for guide sequence used in CRISPR/Cas9 system

Primers	Sequence
PX330-KOwt-1-F	5'-CACCGTCACCACTCCAGGCCTTATT-3'
PX330-KOwt-1-R	5'-AAACAATAAGGCCTGGAGTGGTGAC-3'
PX330-KOwt-2-F	5'-CACCGAGGACTGTAGTACTAAAGA-3'
PX330-KOwt-2-R	5'-AAACTCTTTAGTACTACAGTCCTC-3'

Supplementary Table 7. Oligonucleotides for guide sequence used in CRISPR/Cas9 system.
The sequences of oligonucleotides for constructing guide sequence cloned into pX330 plasmid are described.
